# Effects of Apnoea versus Normal Breathing on Physiological Responses during High-Intensity Interval Training in Swimming

**DOI:** 10.5114/jhk/195587

**Published:** 2025-05-26

**Authors:** Pinelopi Liapaki, Helen Soultanakis, Ioannis Kalomenidis, Vicente Javier Clemente-Suárez, Stamatis Mourtakos, Spyros Zakynthinos

**Affiliations:** 1 Medical School, National and Kapodistrian University of Athens, Athens, Greece.; 2 Human and Evolutionary Biology Section, Department of Biological Sciences, University of Southern California, Los Angeles, California, USA.; 3 1st Department of Critical Care and Pulmonary Medicine, School of Medicine, National and Kapodistrian University of Athens, Greece.; 4 Faculty of Medicine, Health and Sports, Universidad Europea de Madrid, Villaviciosa de Odón, 28670 Madrid, Spain.; 5 Research Group in Culture, Education, and Society, University of the Coast, Barranquilla, Colombia.; 6 School of Medicine, Department of Physiology, National and Kapodistrian University of Athens, Athens, Greece.; 7 School of Medicine, Department of Critical Care and Pulmonary Medicine, National and Kapodistrian University of Athens, Athens, Greece.

**Keywords:** sprint, acid-base, aquatic sports

## Abstract

The objective of this research was to examine the impact of conventional breathing versus apnoea technique on acid-base equilibrium, physiological reactions, and performance throughout high-intensity interval training sessions in swimming. Two groups of sixteen athletes completed 6 x 50-m intervals of freestyle swimming with normal breathing and apnoea at maximum intensity, with a 1-min rest interval. Capillary blood gasses (pH, PCO_2_, PO_2_, HCO_3_, Hct, Hb) were collected at four measurement time points: 1) at rest, 2) at rest just after the 3^rd^ repetition, 3) at finish, and 4) at the 10^th^ min of recovery. Measured variables included the heart rate (HR) during swimming, lactate (La) concentration and swimming time (t_50_). Uncompensated metabolic acidosis, exhibiting greater prominence during apnoea, was attributed to heightened lactic acidosis under both breathing conditions. Despite experiencing bradycardia, swimmers demonstrated faster completion times during apnoea. In conclusion, during repeated high-intensity short-distance swimming, specifically 50 m of freestyle, apnoea enhances sprint performance without compensating for metabolic acidosis.

## Introduction

Competitive swimming has experienced significant advancements in training methodologies and understanding of physiological responses. One specific technique that has gained attention is dynamic apnoea, where short-distance swimmers incorporate breath-holding for multiple arm stroke cycles during training. This method aims to reduce drag force for biomechanical optimization, thereby enhancing swimming technique and maximizing propulsion (Key et al., 2014; Lemaitre et al., 2010). Additionally, controlled frequency breathing over extended distances improves underwater gliding post-start or turn, resulting in increased velocity (Naemi et al., 2010).

Dynamic apnoea training is noteworthy for its impact on both aerobic and anaerobic capacities. Benefits include increased erythropoietin concentration, elevated hematocrit and hemoglobin levels (Liapaki et al., 2024), expanded lung volumes, and improved muscle buffering capacity through reduced blood acidity and oxidative stress (Schagatay et al., 2007; Woorons et al., 2008). Despite these advantages, there remains limited research on the metabolic disturbances associated with dynamic apnoea. Some studies have highlighted bradycardia during moderate-intensity dynamic apnoea (Andersson et al., 2004; Breskovic et al., 2011) and in high-intensity exercises in trained divers (Overgaard et al., 2006; Rodriguez-Zamora et al., 2012). A significant gap exists in understanding acid-base balance variations during maximum intensity freestyle swimming with normal and apnoeic breathing.

Given these literature deficiencies, our research aimed to investigate the effects of high-intensity interval training on physiological and biochemical variables during normal breathing and apnoea swimming. We conducted measurements at intermediate intervals during 6 x 50-m maximum intensity swims, focusing on variables such as the heart rate (HR), lactate concentration (La), and swimming time (t_50_). This study sought to reveal the influence of apnoea on physiological responses compared to normal breathing and to uncover the nuanced adaptations in elite swimmers and freedivers.

## Methods

### Participants

Two groups of thirty-two (32) athletes, sixteen (16) swimmers (8 men, 8 women) and sixteen (16) freedivers (8 men, 8 women) were recruited for the study. All participants were elite athletes with international and national awards and trained 6–9 times a week. The decision to include freedivers came from a new perspective—their unique background as former champion swimmers transitioning into the specialist field of freediving. Over the past 2–3 years, these athletes had redirected their training efforts toward mastering the complex techniques of freediving, a discipline that relies heavily on prolonged breath-holding abilities and efficient oxygen use. This transition presented an excellent opportunity to compare the physiological adaptations of athletes who once had excelled in the controlled environment of pools with those who throve in the deep sea, where breath duration and oxygen management are paramount. Freedivers underwent rigorous training regimens, engaging in pool sessions 6–9 times a week, which encompassed both swimming drills and freediving-specific exercises conducted along the length of the pool.

The characteristics (mean ± standard deviation) of participants were: VO_2max_ = 53.36 ± 6.51 ml•kg^−1^•min^−1^, age 24.7 ± 3.1 years, body height 175.0 ± 6.0 cm and body mass 68.2 ± 8.6 kg. After being informed in writing and verbally, with every detail about the purpose of the research, the experimental procedures and the possible risks involved, participants signed informed consent. All procedures were approved by the Institutional Ethics Committee of the Medical School, National and Kapodistrian University of Athens, Evaggelismos hospital, Greece (protocol code: 3370; approval date: 29 January 2021) and the study was conducted following the principles of the Declaration of Helsinki.

### Measures

#### Regulation of Breathing Processes

The ergospirometer (Ultima Series, MedGraphics, USA) was used to measure the dynamic lung volumes and capacities (FEF_25–75%_: expiratory flow at 50% of expiration, FEFmax: maximum expiratory flow, FEV_1_/FVC: ratio of the two previous volumes, and FEV_1_: forced expiratory volume in 1 s).

In order to capture the resting tidal volume for five normal breathing cycles, the participant in the forced expiratory vital capacity (FVC) test breathed calmly and normally through the mouthpiece while obstructing nasal air exchange using a nasal pressurizer. The participant was instructed to execute a maximal inhalation and a maximal dynamic exhalation until the lungs were empty at the conclusion of the fifth calm exhalation.

The test was administered three times, with a variation of less than 5% between attempts, in accordance with the worldwide guidelines set forth by the American Lung Association (ATS 2011). Every five minutes or more was spent in between each spirometry measurement.

#### Assessment of VO_2max_ and High-Intensity Exercise

Participants' VO_2max_ was evaluated using the Bruce procedure, which involved progressively raising the treadmill's speed and slope until the test's variables were satisfied. Based on their VO_2max_ measurements, the intensity of the exercise session was established for each participant individually. Running on a treadmill for three minutes at progressively greater speeds—corresponding to fifty-five, sixty-five, and eighty- five percent of the participants' maximum oxygen uptake—constituted the high-intensity exercise session. Participants started the first-minute running at 55% of their VO_2max_ and progressed without stopping to 65% and 85% of their VO_2max_ for the second and third minutes, respectively. For the exercise trials, a mechanically operated treadmill with % grade and speed modifications were utilized (TECHNOGYM, Italy).

#### Process of Obtaining Blood Gasses

**Figure 1:**
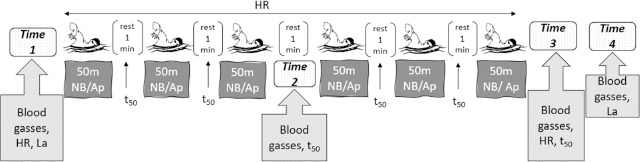
Experimental procedures of the study for the two sessions performed on different days. Participants swam 6 x 50 m freestyle with Normal Breathing (NB) and 6 x 50 m freestyle with Apnoea (Ap). Measured variables included the heart rate (HR), blood gasses (pH, PCO_2_, PO_2_, HCO_3_, Hct, Hb), lactate (La) concentration and swimming time (t_50_).

Capillary blood gasses were measured during the tests at four different time points: before swimming (time 1), immediately after the third repetition (time 2), at the end (time 3), and after ten minutes of recovery (time 4). These blood gasses included potential of hydrogen (pH), partial pressure of carbon dioxide (PCO_2_), partial pressure of oxygen (PO_2_), bicarbonate (HCO_3_), hematocrit (Hct), and hemoglobin (Hb) (Figure 1). The analysis was performed using the GASTAT-602i capillary blood gas analyzer (Techno Medica, USA). Before every blood collection, the finger region was heated with a hot water container to 37°C to guarantee arterialization of the sample (Linderman et al., 1990). Before drawing blood, the participants' skin was always lightly cleansed and treated with 96% alcohol to serve as an antiseptic. Skilled medical professionals used a scriber to take a capillary blood sample from the participant's upper extremity fingertip, which they then transferred into a 130μl capillary blood gas tube.

#### Measurement of Lactate (La) Concentration, Swimming Time (t50), and the Heart Rate (HR)

A wireless, waterproof Polar Pro strap heart rate monitor (Polar, H10, Finland) was used to continually track athletes' heart rates while they were swimming. On the basis of mean values, additional data analysis was performed (Figure 1). An electronic stopwatch (Casio, HS80tw) was used to measure swimming time (t_50_) under all experimental conditions. The average of six repetitions for each breathing condition was employed in analyses (Figure 1).

Lactate (La) concentration was evaluated during each test (Figure 1) at rest before swimming and eight minutes after recovery because the peak value in high-intensity exercise occurs five to ten minutes after the cessation of exercise (Hermansen and Stensvold, 1972). A sample of 10 microliters of capillary blood was obtained from the athletes' fingertips and placed into a portable lactic acid analyzer (Lactate Scout 4, Leipzig, Germany). It has been demonstrated that, in comparison to a laboratory analyzer, the portable analyzer demonstrates good validity and reliability (Tanner et al., 2010) (Figure 1).

### Design and Procedures

Every measurement was performed in a 50-m outdoor swimming pool with the water temperature of 27°C. Participants visited the lab four times. The first stage took place over two days. The first day's main objectives were to get each participant acquainted with the measuring tools and the research setting. Additionally, anthropometric measurements were taken, respiratory control was assessed, and static apnoea was evaluated. A spirometer, an oscilloscope, and a Polar heart rate monitor were used to track the participants' relaxation state for 10 min while they were seated. All of this time, data were continuously recorded. The maximum static apnoea test was carried out with the participant's body and face submerged in water, and the duration was registered, following a 30-min rest interval.

The assessment of respiratory capacity was the main goal of the second day of preliminary testing. A simple treadmill test was performed, increasing the speed and the percentage grade until the VO_2max_ requirements of the Bruce protocol were met.

The primary experimental protocol consisted of two identical sessions conducted in a cross-over design with a 24-h interval between each trial, one week following the preliminary phase. Participants swam 6 x 50 m freestyle with 1-min rest intervals in between, with normal breathing during the first session (i.e., one breath every 3 arms strokes) and with apnoea during the second session. All athletes adhered to a calculated dietary plan the day before and the day of the test. Additionally, they received instructions not to engage in any strenuous exercise 24 h before each experimental session. We postulated that apnoea might have a greater impact on physiological reactions leading to decreased athletes' performance compared to normal breathing.

### Statistical Analysis

For every variable that was recorded, mean values, standard deviations, and/or standard errors were computed. The Kolmogorov-Smirnov test was used to determine whether the data had a normal distribution. The Mauchly’s test confirmed sphericity. The Greenhouse-Geisser approach was used to alter the significance of F-ratios when the assumption of sphericity was not met. A repeated measures ANOVA with a within-two factor (time of measurement and breathing condition) was utilized to compare the variations of all dependent variables that were evaluated twice or more. At each time point, the difference between apnoea and regular breathing was compared using a paired samples *t*-test.

The primary dependent variables analyzed using ANOVA were the heart rate (HR), swimming time (t_50_), and blood gasses (pH, PCO_2_, PO_2_, HCO_3_, Hct, and Hb). Using Cohen's *f*, the effect size for the F-statistics was calculated. The effect size was considered small if the absolute value of Cohen’s *f* was less than 0.10, medium if it was between 0.10 and 0.25 and large if it was greater than 0.25 (Cohen, 1988). Additionally, the pooled standard deviation was used as the denominator in Cohen's *d* to determine the effect size for paired comparisons. The effect size was considered small if the absolute value of Cohen’s *d* was less than 0.20, medium if it was between 0.20 and 0.50 and large if it was greater than 0.50 (Cohen, 1988). In every statistical analysis, the significance level was established at 5% (*p* < 0.05). Statistical software SPSS (Statistical Product and Service Solutions) version 25 was used to conduct all statistical analyses.

## Results

### Respiratory Control, Static Apnoea, VO_2max_

Respiratory variables are presented with mean ± standard deviations (SD): FVC = 4.89 ± 1.05, FEV_1_ = 4.13 ± 0.76, FEV_1_/FVC = 87.13 ± 4.44, FEF_25–75%_ = 4.85 ± 0.76, FEF_max_ = 9.65 ± 3.02. The mean static apnoea had duration of 4.30 ± 0.30 s and the mean aerobic capacity (VO_2max_) was 53.36 ± 6.51 ml•kg^−1^•min^−1^.

### Blood gasses

#### pH Levels

**Figure 2:**
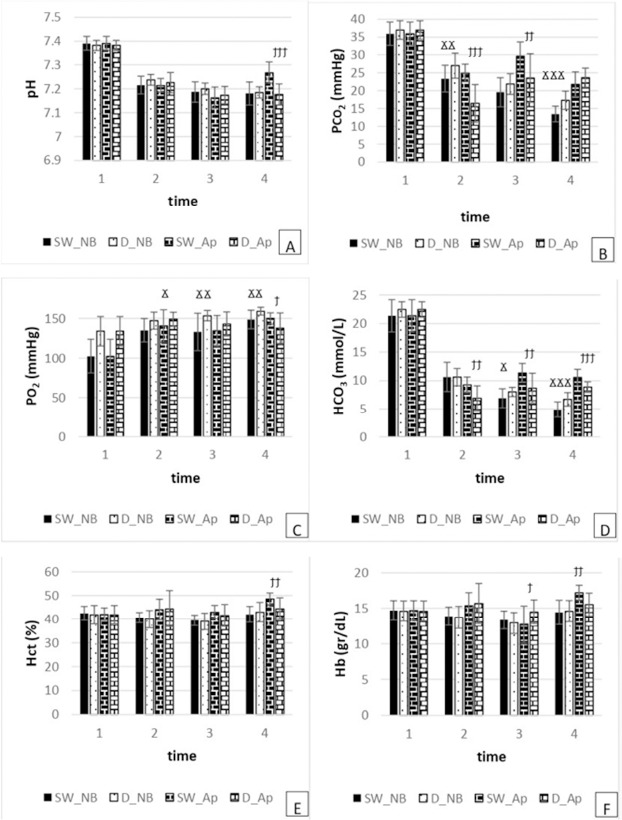
Measured variables in Normal Breathing (NB) and Apnoea (Ap) Swimmers (SW) at each time point (*time 1*: at rest before swimming, *time 2*: at rest just after the 3^rd^ repetition, *time 3*: at finish, *time 4*: at recovery). Significant difference in normal breathing (NB) between swimmers (SW) and freedivers (D) at each time point, ^Ϫ^ *p < 0 .05*, ^ϪϪ^ *p < 0.01*, ^ϪϪϪ^ *p < 0.001*. Significant difference in apnoea (Ap) between swimmers (SW) and freedivers (D) at each time point, ^ϯ^ *p < 0.05*, ^ϯϯ^ *p < 0.01*, ^ϯϯϯ^ *p < 0.001*

*Normal Breathing:* No significant pH differences were found between swimmers and freedivers at any time point (*p* > 0.05). A trend towards significance was noted at the 2^nd^ time point (*p* = 0.054), while the 3^rd^ (*p* = 0.273) and 4^th^ (*p* = 0.749) time points showed no significant differences (Figure 2A).

*Apnoea:* Significant differences in mean pH were observed only at the 4^th^ time point (*p* = 0.000). No significant differences were found at the 2^nd^ *(p* = 0.388) and 3^rd^ (*p* = 0.467) time points (Figure 2A).

#### PCO_2_ Levels

*Normal Breathing:* Significant differences were noted between swimmers and freedivers at the 2^nd^ (*p* = 0.008) and 4^th^ (*p* = 0.000) time points, with swimmers exhibiting lower PCO_2_ levels. No significant difference was observed at the 3^rd^ time point (*p* = 0.076) (Figure 2B).

*Apnoea:* Significant differences in mean PCO_2_ were found at the 2^nd^ (*p* = 0.000) and 3^rd^ (*p* = 0.004) time points, with swimmers showing higher levels than freedivers. No significant difference was observed at the 4^th^ time point (*p* = 0.106) (Figure 2B).

#### PO_2_ Levels

*Normal Breathing:* Swimmers had significantly lower PO_2_ levels compared to freedivers at all observed time points: 2^nd^ (*p* = 0.013), 3^rd^ (p = 0.004), and 4^th^ (*p* = 0.004) (Figure 2C).

*Apnoea:* Significant differences in mean PO_2_ levels were observed at the 4^th^ time point (*p =* 0.027), with swimmers showing higher PO_2_ levels. No significant differences were found at the 2^nd^ (*p* = 0.141) and 3^rd^ (*p* = 0.169) time points (Figure 2C).

#### HCO_3_ Levels

*Normal Breathing:* Significant differences were observed at the 3^rd^ (*p* = 0.027) and 4^th^ (*p* = 0.000) time points, with swimmers showing lower HCO_3_ levels compared to freedivers. No significant difference was observed at the 2^nd^ time point (*p* = 0.960) (Figure 2D).

*Apnoea:* Significant differences were noted at all time points: 2^nd^ (*p* = 0.001), 3^rd^ (*p* = 0.002), and 4^th^ (*p* = 0.000), with swimmers exhibiting higher HCO_3_ levels compared to freedivers (Figure 2D).

### Hematocrit (Hct) Levels

*Normal Breathing:* No significant Hct differences were found between swimmers and freedivers at any time point: 2^nd^ (*p* = 0.562), 3^rd^ (*p* = 0.645), and 4^th^ (*p* = 0.505) (Figure 2E).

*Apnoea:* Significant differences were observed only at the 4^th^ time point (*p* = 0.003), with swimmers showing higher Hct levels compared to freedivers. No significant differences were noted at the 2^nd^ (*p* = 0.996) and 3^rd^ (*p* = 0.213) time points (Figure 2E).

### Hemoglobin (Hb) Levels

*Normal Breathing:* No significant differences were found between swimmers and freedivers at the 2^nd^ (*p* = 0.741), 3^rd^ (*p* = 0.366), or 4^th^ (*p* = 0.762) time points (Figure 2F).

*Apnoea:* Significant differences were observed at the 3^rd^ (*p* = 0.029) and 4^th^ (*p* = 0.002) time points, with swimmers showing lower Hb levels at the 3^rd^ time point and higher levels at the 4^th^. No significant difference was found at the 2^nd^ time point (*p* = 0.721) (Figure 2F).

Given the advantages and the latest recommendations of the British Thoracic Society, capillary blood gas should replace arterial blood gas for the investigation and monitoring of respiratory and metabolic disorders (Richter et al., 2014).

### Heart Rate (HR), Swimming Time (t50) and Lactate (La) Concentration

#### Heart Rate (HR)

**Figure 3:**
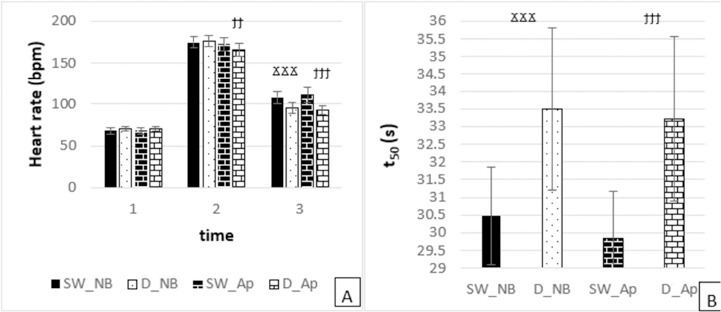
Measured variables in normal breathing (NB) and apnoea (Ap) swimmers (SW) at each time point (*time 1*: at rest before swimming, *time 2*: during swimming, *time 3*: 1^st^ min of recovery). Significant difference in normal breathing (NB) between swimmers (SW) and freedivers (D) at each time point, ^Ϫ^ *p < 0.05*, ^ϪϪ^ *p < 0.01*, ^ϪϪϪ^ *p < 0.001*. Significant difference in apnoea (Ap) between swimmers (SW) and freedivers (D) at each time point, ^ϯ^ *p < 0.05*, ^ϯϯ^ *p < 0.01*, ^ϯϯϯ^ *p < 0.001*

*Normal Breathing:* Significant differences in the mean HR were observed between swimmers and freedivers at the 3^rd^ time point, with swimmers showing a higher HR (*p* = 0.000). No significant difference was noted at the 2^nd^ time point (*p* = 0.572) (Figure 3A).

*Apnoea:* Significant differences in mean HR levels were found between swimmers and freedivers at both the 2^nd^ (*p* = 0.019) and 3^rd^ (*p* = 0.000) time points, with swimmers exhibiting a higher HR compared to freedivers (Figure 3A).

### Swimming Time (t_50_)

*Normal Breathing:* Swimmers had a significantly lower mean t_50_ compared to freedivers (*p* = 0.000) (Figure 3B).

*Apnoea:* Similarly, under apnoeic conditions, swimmers demonstrated a significantly lower mean t_50_ compared to freedivers (*p* = 0.000) (Figure 3B).

#### Lactate (La) Concentration

**Figure 4:**
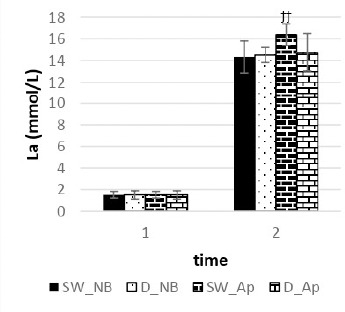
Measured variable in normal breathing (NB) and apnoea (Ap) swimmers (SW) at each time point (*time 1*: at rest before swimming, *time 2*: at 8 min of recovery). Significant difference in normal breathing (NB) between swimmers (SW) and freedivers (D) at each time point, ^Ϫ^ *p < 0.05*, ^ϪϪ^ *p < 0.01*, ^ϪϪϪ^ *p < 0.001*. Significant difference in apnoea (Ap) between swimmers (SW) and freedivers (D) at each time point, ^ϯ^ *p < 0.05*, ^ϯϯ^ *p < 0.01*, ^ϯϯϯ^ *p < 0.001*

*Normal Breathing:* No significant differences in mean blood lactate concentration were observed between swimmers and freedivers (*p* = 0.662) (Figure 4).

*Apnoea:* Significant differences in mean blood lactate concentration were found, with swimmers showing higher lactate values compared to freedivers (*p* = 0.003) (Figure 4).

## Discussion

By juxtaposing the physiological profiles of elite swimmers with those of freedivers, this study aimed to elucidate the divergent adaptive pathways of these athletes. Swimmers primarily rely on the aerobic metabolism and efficient oxygen transport to sustain high-intensity efforts, whereas freedivers optimize oxygen utilization and tolerate hypoxic conditions through rigorous breath-hold training. Through meticulous data collection and analysis in real-world aquatic environments, we investigated the nuanced physiological responses to underwater immersion, highlighting how each discipline shapes the athletes' bodies.

The study's findings offer valuable insights into the physiological responses of swimmers and freedivers under both normal breathing and apnoeic conditions (Liapaki et al., 2024), which are crucial for optimizing training regimens and performance in aquatic sports (Wilson et al., 2020). Notably, data were collected during swimming sessions, recovery phases, and intervals between repeated distances, emphasizing the significance of our results. Among these, the identification of uncompensated metabolic acidosis caused by elevated lactate concentrations post-exercise stands out (Liapaki et al., 2024).

During normal breathing, no significant differences in mean pH levels were found between swimmers and freedivers at any observed time point, despite a near-significant trend at the 2^nd^ time point (*p* = 0.054). This indicates a comparable acid-base balance between the two groups, aligning with prior research (Jones et al., 2020; Smith et al., 2018).

In contrast, a significant disparity in mean pH levels was observed under apnoeic conditions, with swimmers exhibiting higher pH levels at the 4^th^ time point (*p* = 0.000). This difference suggests divergent responses to breath-holding, likely influenced by variations in apnoeic capabilities and physiological adaptations. Our findings corroborate existing literature on the impact of breath-holding on acid-base balance and metabolic responses among aquatic athletes (Brown et al., 2021; Jackson et al., 2019).

Our analysis revealed significant disparities in the mean partial pressure of carbon dioxide (PCO_2_) levels between swimmers and freedivers under both normal breathing and apnoeic conditions. During normal breathing, swimmers exhibited significantly lower PCO_2_ levels compared to freedivers at the 2^nd^ (*p* = 0.008) and 4^th^ (*p* = 0.000) time points, indicating superior CO_2_ elimination efficiency (Brown et al., 2021; Smith et al., 2018). At the 3^rd^ time point, no significant difference was observed (*p* = 0.076), suggesting comparable respiratory dynamics.

During breath-holding, swimmers showed significantly higher PCO_2_ levels at the 2^nd^ (*p* = 0.000) and 3^rd^ (*p* = 0.004) time points, indicating greater CO_2_ retention capacity (Jackson et al., 2019, 2020). By the 4^th^ time point, PCO_2_ levels converged between the groups (*p* = 0.106), reflecting normalization of respiratory responses.

Pronounced hypocapnia followed swimming with regular respiration, whereas a milder form was observed after apnoea swimming. This suggests that swimmers rapidly acclimatized to apnoea, reducing hypocapnia severity. These findings highlight distinct respiratory adaptations and underscore the importance of tailored training strategies for CO_2_ management in aquatic athletes (Wilson et al., 2020).

Additionally, significant differences in mean partial pressure of oxygen (PO_2_) levels were noted between swimmers and freedivers under both normal breathing and apnoeic conditions. During normal breathing, swimmers had significantly lower PO_2_ levels at all observed time points: 2^nd^ (*p* = 0.013), 3^rd^ (*p* = 0.004), and 4^th^ (*p* = 0.004) (Brown et al., 2021; Smith et al., 2018). This suggests differences in oxygen uptake or utilization efficiency.

During breath-holding, significant differences in PO_2_ levels were observed at specific time points. At the 4^th^ time point, swimmers had significantly higher PO_2_ levels (*p* = 0.027), indicating enhanced oxygen retention or delivery mechanisms (Jones et al., 2020). However, no significant differences were noted at the 2^nd^ (*p* = 0.141) and 3^rd^ (*p* = 0.169) time points, indicating a nuanced interplay of physiological responses to breath-holding across the groups.

Our study revealed significant differences in mean bicarbonate (HCO_3_) levels between swimmers and freedivers under both normal breathing and apnoeic conditions. During normal breathing, swimmers had lower HCO_3_ levels at the 3^rd^ (*p* = 0.027) and 4^th^ (*p* = 0.000) time points, indicating differences in acid-base balance regulation (Brown et al., 2021; Wilson et al., 2020). No significant difference was found at the 2^nd^ time point (*p* = 0.960), suggesting comparable responses in early stages of the test.

During apnoea, swimmers exhibited significantly higher HCO_3_ levels at all time points (2^nd^: *p* = 0.001, 3^rd^: *p* = 0.002, 4^th^: *p* = 0.000), indicating distinct metabolic adaptations (Jones et al., 2020).

Research on respiratory responses in aquatic athletes shows mixed results. Swimmers display reduced respiratory response to hypercapnia and hypoxia compared to controls (Arce-Álvarez et al., 2021; Ohkuwa et al., 1980). Conversely, elite breath-hold divers showed no significant differences in respiratory and cardiovascular responses to hypoxia and hypercapnia compared to controls (Breskovic et al., 2010a; Dujic et al., 2008). After endurance training, divers normalized peripheral chemosensitivity regulation.

Non-apnoea training may not affect breath-holding or chemoreflex response, but the impact of apnoea training on chemosensitivity control warrants further investigation (Arce-Álvarez et al., 2022).

The spleen, a key organ in the body's defense and hematopoietic system, stores significant amounts of oxygenated blood, particularly in elite divers (Prassopoulos et al., 1997). Splenic contraction releases oxygenated blood into systemic circulation, maintaining oxygen saturation and hemoglobin concentration during prolonged apnoea or brief hypoxia (Bakovic et al., 2003; Pernett et al., 2021).

Splenic contraction, largely driven by sympathetic nerve activity, increases hematocrit during hypoxia, exercise, and stress (Bakovic et al., 2013; Stewart et al., 2002).

Our study revealed no significant differences in mean hematocrit (Hct) levels between swimmers and freedivers during normal breathing at any observed time point (2^nd^: *p* = 0.562, 3^rd^: *p* = 0.645, 4^th^: *p* = 0.505), indicating similar blood volume and oxygen-carrying capacity (Smith et al., 2018; Wilson et al., 2020). During apnoea, however, swimmers had significantly higher Hct levels at the 4^th^ time point (*p* = 0.003), suggesting different haematological adaptations to breath-hold stress (Jackson et al., 2019; Wilson et al., 2020). No significant differences were found at the 2^nd^ (*p* = 0.996) and 3^rd^ (*p* = 0.213) time points, indicating comparable responses in early breath-holding stages.

Mean hemoglobin (Hb) levels during normal breathing showed no significant differences between the groups at any time point (2^nd^: *p* = 0.741, 3^rd^: *p* = 0.366, 4^th^: *p* = 0.762), suggesting similar oxygen-carrying capacity (Smith et al., 2018; Wilson et al., 2020). In contrast, during apnoea, significant differences in Hb levels emerged at specific times. At the 3^rd^ time point, swimmers had significantly lower Hb levels than freedivers (*p* = 0.029), indicating potential differences in oxygen utilization or metabolic responses (Jackson et al., 2019). By the 4^th^ time point, swimmers showed significantly higher Hb levels (*p* = 0.002), suggesting possible compensatory mechanisms to prolonged apnoea stress (Brown et al., 2021). No significant differences were observed at the 2^nd^ time point (*p* = 0.721), indicating comparable initial Hb levels.

Schagatay et al. (2012) found that elite swimmers with greater splenic emptying experienced longer apnoea duration, suggesting that the chemoreceptor-sympathetic-spleen pathway could be crucial for maintaining oxygen supply during swimming (Arce-Álvarez et al., 2022). These findings highlight the dynamic respiratory and haematological responses in aquatic athletes and emphasize the need for further research to understand the mechanisms behind these differences and their implications for performance and health.

During normal breathing, significant differences in the heart rate (HR) were noted between the groups at specific times, with swimmers showing a significantly higher HR at the 3^rd^ time point (*p* = 0.000), suggesting different cardiovascular responses or autonomic regulation (Smith et al., 2018). No significant difference was observed at the 2^nd^ time point (*p* = 0.572), indicating similar early cardiovascular responses.

During apnoea, significant differences in mean HR levels were observed, with swimmers displaying higher HRs at both the 2^nd^ (*p* = 0.019) and 3^rd^ (*p* = 0.000) time points, indicating heightened sympathetic activation or cardiovascular stress during breath-holding (Jones et al., 2020).

Each turn of the head for breathing disrupts a swimmer's hydrodynamic position in freestyle swimming, increasing resistance and decreasing efficiency (Counsilman, 1955; Maiello et al., 1998; Morais et al., 2024; Scott et al., 2024). This disadvantage is particularly significant in sprint events, where apnoea offers a performance advantage, as confirmed by our results.

During normal breathing, swimmers had significantly lower swimming times compared to freedivers (*p* = 0.000), indicating superior aerobic capacity and swimming efficiency (Liapaki et al., 2024; Smith et al., 2018; Wilson et al., 2020). Similarly, under apnoeic conditions, swimmers exhibited significantly lower t50 values than freedivers (*p* = 0.000), reflecting better breath-holding capabilities and oxygen utilization (Brown et al., 2021; Jackson et al., 2019; Liapaki et al., 2024).

Performance was enhanced during apnoea for both groups, with swimmers outperforming freedivers. This suggests that, in swimmers, better performance with an unchanged heart rate during apnoea indicates that oxygen delivery to muscles is not directly linked to performance (Liapaki et al., 2024). In freedivers, increased performance with bradycardia suggests a similar dissociation (Konstantinidou and Soultanakis, 2016). These results imply that swimmers have a greater adaptive capacity to apnoea, achieving better times at the same heart rate (Liapaki et al., 2024), while freedivers exhibit the diving reflex more prominently.

Contrary to research suggesting that apnoea bradycardia reduces performance or leaves it unchanged with an increased heart rate (Guimard et al., 2017b), our findings suggest that performance in 50-m apnoea tests is achievable with high apnoea and aerobic capacities (static apnoea 4.30 ± 0.30 s; VO_2max_ = 53.36 ± 6.51 ml•kg^−1^•min^−1^).

During normal breathing, no significant differences in mean blood lactate concentrations were found between swimmers and freedivers (*p* = 0.662), suggesting similar metabolic responses and lactate clearance rates (Smith et al., 2018; Wilson et al., 2020). However, during apnoea, swimmers showed significantly higher lactate concentrations (*p* = 0.003), indicating an increased anaerobic metabolism and lactate production due to intense peripheral vasoconstriction and reduced oxygen transport (Brown et al., 2021; Jackson et al., 2019; Liapaki et al., 2024; Woorons et al., 2008, 2024).

These findings underscore the distinct metabolic and physiological responses to breath- holding in swimmers and freedivers, highlighting the need for further research to elucidate the underlying mechanisms and their implications for performance and training strategies in aquatic sports.

Overall, our study adds to the growing body of literature on aquatic athletes' physiological responses, emphasizing the importance of understanding these adaptations to optimize training and performance under competitive conditions.

## Conclusions

The primary finding of this study was that exercise-induced lactic acidosis led to uncompensated metabolic acidosis during both normal breathing and apnoea-induced swimming. Despite improved performance in all repeated distances, acidity levels were more pronounced during apnoea, contradicting our hypothesis that physiological and metabolic reactions would impair performance. Swimmers began with elevated acidosis during apnoea repeats, but showed notable performance improvement, suggesting enhanced H^+^ ion control despite increased metabolic disruption, as indicated by higher lactate concentrations.

There was no significant difference in heart rates between normal breathing and apnoea swimming, indicating that the diving response did not mitigate exercise-induced tachycardia, as bradycardia was absent in swimmers. This raises concerns about potential anoxia in critical organs if heart rate responses are solely used for evaluation. Conversely, bradycardia was observed in freedivers during apnoea. A significant aspect of the apnoea response involved forceful spleen contraction, increasing hemoglobin and hematocrit levels, thereby enhancing the bloodstream's capacity to release stored erythrocytes and maintain oxygen availability, improving performance during apnoea.

In conclusion, this study bridges competitive swimming and freediving, revealing remarkable physiological adaptations in aquatic athletes. Through rigorous experimentation and analysis, we deepened our understanding of the physiological intricacies underlying elite athletic performance in aquatic environments. Our findings should inspire future generations of aquatic athletes to explore new frontiers of excellence and discovery.

## Perspective

The present study revealed significant findings on the effects of apnoea on athletes’ performance. However, these results may have practical implications that were not fully developed in the original paper. Coaches and athletes could incorporate apnoea into their training programs to improve endurance and anaerobic capacity. Incorporating apnoea techniques, based on the results of this study, could optimize training programs, especially in sports such as swimming, artistic swimming, freediving, underwater rugby, hockey and running.

Apnoea training improves performance in the 50-m freestyle by training the anaerobic metabolism. However, it must be strictly supervised by coaches, even up to 10 min after its cessation, due to the continued risk of metabolic acidosis, posing serious health concerns.

Additionally, our research highlights the potential for interdisciplinary collaboration between swimming and freediving communities, fostering innovation and advancement in both disciplines.

Although the study focuses on high-level athletes, the findings may have implications for other populations, such as amateur swimmers or athletes in sports that require breath control, such as martial arts or freediving. It is important to consider how these results can be adapted and applied to different groups, taking into account the specificities and objectives of each population.

It is necessary to further investigate the long-term effects of apnoea training, to evaluate its effectiveness in different types of athletes and to incorporate different apnoea techniques into training programmes in order to increase performance and safety.

In addition, the study could further examine the dynamics of recovery after apnoea compared to normal breathing. Understanding the body's long-term adaptation to apnoea, as well as the potential risks that may arise, is critical to the safe and effective incorporation of this technique into training.
